# Supplementary material for: The impact of childcare availability on maternal employment: Evidence from Czech municipalities
Source: PLoS One. 2023 Jul 20;18(7):e0288987. doi: 10.1371/journal.pone.0288987 (PMC10358966; doi:10.1371/journal.pone.0288987)
Supplement: Attachment — Submitted filename: Response to Reviewers.docx [file pone.0288987.s001.docx]

**PONE-D-22-33710**

**The Impact of Childcare Availability on Maternal Employment: Evidence from Czech Municipalities**

**PLOS ONE**

**Reviewer #1**

Dear authors, I am glad I have this opportunity to review your manuscript. My comments are as follows (let me divide my comments according to the structure of your manuscript):

Abstract

- I personally expect that the aim of the study will be exactly defined, but it is missing in the current version

- identify "various sources" you are using for your research

- data used are more than 10 years old, do you consider it as still actual?

*- This is a retrospective study and using older data is quite plausible. Moreover, the lack of kindergarten places persists in the Czech Republic even these days and we believe that our estimates of the impact of childcare availability on maternal employment are informative for the current situation – see the discussion of* *the topicality of our results and the data availability that we added at the end of the Introduction (highlighted text, p. 4).*

- add more information about the methodology (you are presenting partial results without information about the way of their processing/achieving)

- be more specific (...is lower than...; is closer to...)

*- Thank you. We have changed the abstract according to your comments – clearly defined the aim, added specific details about the data used and methodological approach.*

Introduction- the topic is introduced at a good level by using actual literature

Institutional background- additional information about the situation in the Czech Republic is presented at an expected level

Methodology- selection of indicators should be explained (you are working with local kindergarten availability and neighboring kindergarten availability, but why? what kind of indicators are used to capture the availability of kindergartens? show other research/indicators used for a similar purpose to declare what is new in your research

*- We added a paragraph explaining that our indicators of childcare availability are quite commonly used in the literature, while also explaining the differences and the reason for the need of two indicators. See the Methodology section, highlighted text at p. 8.*

- add some numeric information about both indicators used (at least describe them by some moment characteristics)

*- We added moment characteristics to Table 1 and described them in Section 3.1. We also added the description of distribution of these indicators that was originally in Section 4.1., see the highlighted text at p. 8-9 and 10-11.*

- the same problem with the regression model, any explanation for choosing this method is missing (I agree with your choice, but I also expect much more information)

*- We added the reasoning behind the choice of our method to the beginning of Section 3.2 (see the highlighted text at p. 11).*

Results- all tables/figures in section 4.1 are described very well, but there is no idea throughout the whole text- each paragraph starts "XXX presents something...", after this paragraph you are not working with achieved information

*- The content of this entire section was moved to other parts of the manuscript where they are more relevant, mostly to Section 3.1. This is also in line with the comment above that we should add some descriptive statistics about the indicators of kindergarten availability.*

- section 2.1 is much better prepared, the description of the results is divided and interpreted in detail

- an indicator of change in the employment rate and other parameters/indicators should be described in the methodology, not in the results (section 4.3)

*- Thank you, we provide descriptive statistics of the employment rate in the Methodology section now (see highlighted text at p. 7).*

Discussion and conclusion- add information about the limitations of your research

*- We added two paragraphs discussing the limitations of our research to the end of conclusion (see highlighted text at p. 22).*

Formal notes

- add the name of the axis Y (Figure 1, Figure 2)

*- Added.*

I wish you all the best with this manuscript and other ones in the future

**Reviewer #2**

The paper provides estimates of the impact of local public kindergarten availability on the employment rate of Czech mothers of pre-school children. The authors use unique datasets from the Census carried out by the Czech Statistical Office in 2001 and 2011 matched with the data on the capacity of kindergartens in Czech municipalities from the Ministry of Education, Youth and Sports. The estimates are lower than those published previously for the Czech Republic, although the evidence is limited and the methodology is different, while they are comparable to previous estimates for other Southern and Western European countries. In the final part the authors calculate the aggregate effects and fiscal impacts. The paper is definitely technically sound. I appreciate the part explaining identification assumptions and the discussion in the concluding section on why the estimates are different from results for other countries in the region.

I see the discussion on why the estimates are different as the main challenge. I discuss below some related points.

First, the measurement errors described on p. 12 distort the coefficient estimates towards zero. Are the measurement errors responsible for at least part of the difference between their results and those in Lovász and Szabó-Morvai (2017)? I understand the main difference is in methodology as Lovász and Szabó-Morvai (2017) rely on childcare eligibility cutoffs comparing children that reach the age of 3 around the eligibility cuttoff.

- *We added a discussion of the measurement error and explained why we do not think that it causes the difference between our results and results from previous studies to the Conclusion (highlighted text at p.22).*

Second, the generous parental leave in the Czech Republic (pp. 4-5) allows mothers to stay with their children at home beyond 3 years of age, while the state support is less generous in other countries. Could this explain why the estimates are lower?

- *As we mention in the Conclusion (p.21), Lovász and Szabó-Morvai (2017) provide estimates for both Czech Republic and Hungary and their results are very similar for these two countries that differ substantially in their institutional setting (including parental leave, which is shorter in Hungary). Therefore, we argue that generosity of the parental leave system is likely not the most important determinant of these differences.*

Third, the Czech Republic has very large number of self-governed municipalities which are the main providers of kindergarten places. I suspect the typical commuting distance to potential work could be longer than within municipalities and mothers take advantage of kindergartens available near their workplaces. Is it relevant? Considering different level of aggregation could be left for future research.

- *It is of course possible that some parents prefer kindergartens that are close to their workplace even though they are further away from their home. If that is the case, this introduces another measurement error to our neighboring childcare availability measure. Although there are no statistics on this, anecdotal evidence suggests that it is not very frequent case for several reasons. First, children are eligible for child care places only at the municipality they have their registered permanent residence and not at locations where parents work. Also, picking up children is frequently taken care by other members of the family such as grandparents who guide children to after school sport and other activities. This would not work well if childcare facility were located too far from residential address. But anyway, we added this point to our discussion of identification assumptions in Section 3.3 (see the highlighted text at p. 14), thank you for pointing that out.*
- *With our data, the only alternative level of aggregation would be a region, and this would mean going from 6,098 observations to 76, i.e., loosing most of the variation in childcare availability.*

Finally, are the aggregate effects large or small? The calculation relies on parameters and other estimates from Kalíšková et al. (2016). I would appreciate some discussion of robustness of the calculation. I suggest to elaborate on it and mention the aggregate effects in the abstract or remove this part.

- *Thank you, we discussed the methods used to calculate the aggregate effect and its robustness in more detail in Section 4.2 (see highlighted text at p. 19) and added the results concerning the aggregate effects to the abstract.*

Overall, I enjoyed reading the paper. My comments are meant to improve the discussion related to the comparison with previous literature as well as the robustness of their calculation of aggregate impacts.
